# The IMSI procedure improves poor embryo development in the same infertile couples with poor semen quality: A comparative prospective randomized study

**DOI:** 10.1186/1477-7827-9-123

**Published:** 2011-08-29

**Authors:** Katja Knez, Branko Zorn, Tomaz Tomazevic, Eda Vrtacnik-Bokal, Irma Virant-Klun

**Affiliations:** 1 Reproductive Unit, Department of Obstetrics and Gynaecology, University Medical Centre Ljubljana, Ljubljana, Slovenia

## Abstract

**Background:**

Sperm of poor quality can negatively affect embryo development to the blastocyst stage. The aim of this comparative prospective randomized study was to evaluate the role of an intracytoplasmic morphologically selected sperm injection (IMSI) in the same infertile couples included in the programme of intracytoplasmic sperm injection (ICSI) due to their indications of male infertility which had resulted in all arrested embryos following a prolonged 5-day culture in previous ICSI cycles.

**Methods:**

Couples exhibiting poor semen quality and with all arrested embryos following a prolonged 5-day culture in previous ICSI cycles were divided into two groups: *Group 1: IMSI group *(n = 20) with IMSI performed in a current attempt and *Group 2: ICSI group *(n = 37) with a conventional ICSI procedure performed in a current attempt of *in vitro *fertilization. Fertilization rate, embryo development, implantation, pregnancy and abortion rates were compared between current IMSI and conventional ICSI procedures, and with previous ICSI attempts.

**Results:**

The *IMSI group *was characterized by a higher number of blastocysts per cycle than the *ICSI group *(0.80 vs. 0.65) after a prolonged 5-day embryo culture. There was a significantly lower number of cycles with all arrested embryos and cycles with no embryo transfer in the *IMSI group *versus the *ICSI group *(0% vs. 27.0%, p = 0.048). After the transfer of embryos at the blastocyst or morula stage (on luteal day 5) a tendency toward higher implantation and pregnancy rates per cycle was achieved in the *IMSI group *compared to the *ICSI group *(17.1% vs. 6.8%; 25.0% vs. 8.1%, respectively), although not statistically significant. After IMSI, all pregnancies achieved by the blastocyst transfer were normally on-going, whereas after ICSI, two of three pregnancies ended in spontaneous abortion. After IMSI, two pregnancies were also achieved by the morula stage embryos, whereas after the conventional ICSI procedure, embryos at the morula stage did not implant.

**Conclusions:**

The IMSI procedure improved embryo development and the laboratory and clinical outcomes of sperm microinjection in the same infertile couples with male infertility and poor embryo development over the previous ICSI attempts.

## Background

Infertile patients with abnormal sperm parameters display an elevated sperm aneuploidy rate, despite a normal blood cell karyotype [1]. Indications of male infertility, such as elevated follicle-stimulating hormone (FSH) levels, sperm concentration of less than 1 million per ml, and severe teratozoospermia, are significantly associated with higher numbers of aneuploid sperm [2]. Teratozoospermia can be associated with aneuploidy [1]. Increased aneuploidy has been found in both sperm and embryos in couples with severe male infertility and a history of repeated unsuccessful IVF attempts [3]. Impaired sperm DNA integrity such as sperm DNA fragmentation or denaturation has been related to poor semen quality [4] and may negatively affect embryo development and quality [5]. Overall, a negative relationship has been observed between poor quality sperm and embryo development, even before activation of the embryonic genome, suggesting that sperm can affect embryogenesis from a very early stage [6].

The motile sperm organelle morphology examination (MSOME) allows the selection of the best sperm before oocyte microinjection [7,8]. There have been some reports on improved implantation and ongoing pregnancy rates using this sperm selection method, which is used for intracytoplasmic morphologically selected sperm injection (IMSI) [9]. IMSI enables the selection of motile spermatozoa with fine nuclear morphology and without head vacuoles in real time at high magnification (> 6000 ×), which are then injected into oocytes. The most predictive factor of sperm quality is the incidence of vacuoles in the sperm head. Vacuoles appear to be related to sperm DNA damage (fragmentation and/or denaturation) [10,11] and negatively affect human embryo development. The IMSI method seems to improve the clinical outcome of sperm microinjection of oocytes in terms of embryo quality and development, and, consequently, the embryo implantation and pregnancy outcome [7,8,12-15]. The best spermatozoa selected by the MSOME are mostly acrosome-reacted spermatozoa [16]. In spite of the promising publications, the clinical role of the IMSI method and the indications for it have not been well established.

The aim of this prospective study was to evaluate the clinical role of the IMSI procedure in the same couples displaying indications of male infertility and previously expressed poor embryo development after conventional ICSI.

## Methods

### Patients

This prospective study included couples involved in the ICSI programme at the IVF unit of the University Medical Centre Ljubljana, from October 2009 to June 2010. A total of 57 couples, fitting the following inclusion criteria, were compared in the trial: all couples had indications of male infertility with poor semen quality and were characterized by all embryos arrested after prolonged 5-day embryo culture to the blastocyst stage in their previous conventional ICSI attempts. Poor semen quality was characterised by the incidence of teratozoospermia by less than 14% of morphologically normal sperm according to the Strict Kruger Criteria, oligozoospermia by a sperm concentration of less than 20 million per ml and asthenozoospermia by less than 50% of motile sperm according the WHO criteria. We excluded patients with endometriosis and polycystic ovaries and female partners over 42 years old. After having obtained the patients informed consent, couples were randomly allocated to receive the current procedure of sperm microinjection (IMSI or ICSI) using computer generated random numbers (unrestricted randomization list), concealed in sealed, opaque envelopes prepared by a research nurse. All study participants were blinded as regards their treatment assignment for the duration of the study. The following groups were formed: *group 1 *was named *IMSI group *and included 20 couples who would receive a current IMSI procedure, and *group 2 *was named *ICSI group *and included 37 couples who would receive a current conventional ICSI procedure in the same time period. In each group of couples, only one current cycle was performed - IMSI or conventional ICSI.

Both groups were compared in terms of laboratory and clinical outcomes (fertilization, blastocyst, implantation and pregnancy rates) of the current sperm microinjection procedure, IMSI or conventional ICSI. In the *IMSI group*, the laboratory and clinical outcomes of the 20 current IMSI cycles were compared with the laboratory and clinical outcomes of 33 previously performed unsuccessful conventional ICSI cycles in these couples in the same time period. In the *ICSI group*, the current ICSI laboratory and clinical outcomes were compared with the laboratory and clinical outcomes of 46 previously performed unsuccessful conventional ICSI cycles in these couples in the same time period.

### Ovarian stimulation

Controlled ovarian stimulation was performed using GnRH agonist buserelin (Suprefact; Hoechst AG, Frankfurt/Main, Germany) administered from day 22 of the cycle in a daily dosage of 0.6 ml (600pg) s.c. After 14 days, the pituitary desensitization was checked by E_2 _determination and B-mode ultrasound scan. Once the criteria for desensitization were fulfilled (E_2 _≤ 0.05 nmol/l, follicles ≤ 5 mm in diameter and endometrial thickness ≤ 5 mm), ovarian stimulation with a daily dose of 225 IU highly purified urinary FSH (Menopur; Ferring, Switzerland) was started. GnRH agonist administration was continued until hCG administration. hCG (Pregnyl; N.V. Organon, Oss, The Netherlands) in a dose of 10,000 IU was administered when 3 or more follicles reached a diameter of 18 mm. Ultrasound-guided oocyte retrieval was performed 34-36 h after hCG administration.

### IMSI procedure

Each oocyte was put into a droplet of Sperm Preparation Medium (Origio, Denmark) under paraffin oil (Origio, Denmark). The Partner's semen was prepared by centrifugation on a density gradient (100%/40%) of PureSperm (NidaCon, Sweden) followed by the "swim-up" technique. The fraction of sperm after the "swim-up" technique was used to make droplets under paraffin oil.

Up to 2 hours after oocyte retrieval, the morphological selection of sperm and microinjection of oocytes were performed. Sperm were selected in dishes with a glass bottom (GWSt 1000; Will Co., Wells BV, Amsterdam, The Netherlands) and monitored under an inverted microscope with a heated stage equipped with differential interference contrast (DIC) (Nikon ECLIPSE TE2000-S, Japan). Approximately 5 elongated droplets of SpermSlow medium (Origio, Denmark) were placed on a glass dish bottom to immobilize the sperm. A smaller droplet of prepared sperm was placed near each SpermSlow droplet. Then the connections were made between the sperm and the SpermSlow droplets for sperm to swim into the SpermSlow droplets and to bind to the hyaluronate (HA). All droplets were covered with paraffin oil (Origio, Denmark). For observation under 6,000 times magnification, a droplet of immersion oil was inserted underneath the glass dish (under the SpermSlow droplet). One droplet of SpermSlow with bound sperm was monitored by the immersion objective, differential interference contrast (DIC), and Nikon Digital Sight DS-Ri1 Camera. The single (mature) sperm which bound to the HA and had the best morphology was chosen, aspirated in the microinjection pipette, scored in three dimensions, and evaluated according to the morphology and head vacuoles at 6,000 times magnification.

### Sperm selection criteria

The scoring system for the evaluation and selection of the injected spermatozoa was based on the following classification of Cassuto and his co-workers [14]. In each spermatozoon, three different parameters were evaluated: head shape and dimensions in both axes (normal head: an oval head shape with a regular outline, with a length from 3 to 5 μm, and with a width from 2 to 3 μm according to the WHO criteria), head vacuoles (number and dimensions) and head base normality (normal is a U-shape). The head was scored as follows: 1 point for normality in two or one axes, and 0 points for abnormalities in both axes. No vacuoles in the head or a single vacuole with a small diameter was scored with 1 point; otherwise, the score was 0 points. The normal shape of the sperm head basis was scored with 1 point and abnormal with 0 points. The following formula for the classification of spermatozoon was used:

Score of spermatozoa=(2×Head)+(3×Vacuole)+(Base)

**Figure 1:**
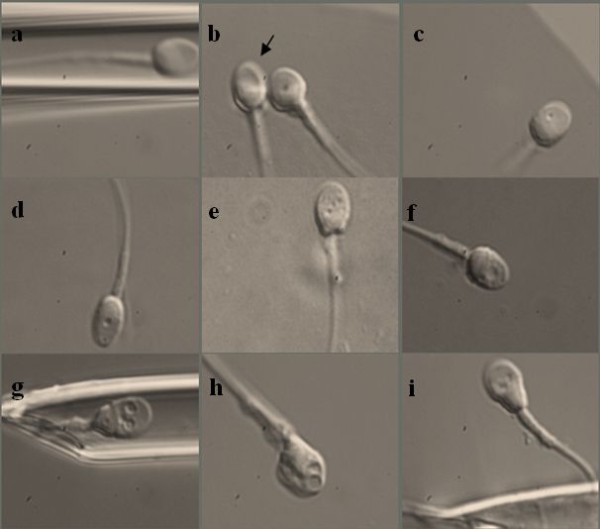
**Classification of spermatozoa selected at 6,000 × magnification into 3 different categories**. Class I - spermatozoa of good quality, Class II - spermatozoa of worse quality, and Class III - spermatozoa of poor quality. *Legend*: a,b,c - spermatozoa of Class I; d,e,f - spermatozoa of Class II; g,h,i - spermatozoa of Class III.

The range for a pre-injected spermatozoon varied between 0 and 6. Spermatozoa were classified into three classes according to this formula (Figure 1): Class I: high-quality spermatozoa with a calculated score of 4 to 6; Class II: medium-quality spermatozoa with a calculated score of 1 to 3; Class III: low-quality spermatozoa with a calculated score of 0. In each patient we tried to select the best sperm for oocyte microinjection. Sperm of class III were not selected for injection into the oocytes. The selected spermatozoa of Class I and Class II were transferred into fresh droplet of Sperm Preparation Medium covered with paraffin oil in a plastic tissue culture dish (Nunc 153066), and were microinjected into the oocytes without the use of polyvinylpyrrolidone (PVP), as described elsewhere [17].

### Conventional ICSI procedure

The sperm selection for microinjection was performed at a magnification of 200 ×-400 ×. Sperm with severe head shape defects clearly seen under the magnification (pin, amorphous, tapered, round, and multinucleated head) were excluded from microinjection into the oocyte. No sperm selection based on MSOME was performed for conventional ICSI.

### Embryo culturing and grading

After microinjection, the oocytes were washed and transferred into the Fertilization Medium (Cook, Australia). The fertilization of the oocytes was checked the next day, 16-18 hours after microinjection. The zygotes were considered to be normal when they had 2 pronuclei and 2 polar bodies, and abnormal when they did not develop further (non-cleaved zygotes) or were triploid (with 3 pronuclei). All embryos developed from the normal zygotes were cultured in the sequential media (Cook, Australia) to the blastocyst stage. On days 2 and 3, they were cultured in the Cleavage Medium and on days 4 and 5 in the Blastocyst Medium. On day 5, embryos were classified according to their developmental stage into advanced embryos - blastocysts, expressing the inner cell mass (ICM), trophectoderm (TE), and blastocoel cavity, and arrested embryos which arrested at the compacted morula stage or stages with lower numbers of cells (low-cell embryos). In each blastocyst, the developmental stage (1-early, 2-developing, 3-developed, 4-expanded, 5-hatching and 6-hatched), the morphology of the ICM (A-good: many cells, tightly packed; B-worse: several cells, loosely grouped; C-bad: very few cells) and the morphology of the TE (A-good: many cells, forming a cohesive layer; B-worse: few cells, forming a loose epithelium; C-bad: very few large cells) was evaluated based on the criteria established by Gardner et al. (2000) [18]. *Good blastocyst quality *was supposed to be at a developed or more advanced stage and with good morphology of ICM and TE, *worse blastocyst quality *at a developed or more advanced stage and with good morphology of only one structure (ICM or TE), and *poor blastocyst quality *showing abnormal morphology of both structures - ICM and TE; very poor blastocyst quality was considered to be at an early stage of development and with abnormal morphology of both structures - ICM and TE (usually combined with the signs of degeneration). At most, 2 embryos at the blastocyst stage were transferred into the uterus on day 5 by the TDT catheter set (CCD, Neuilly, France); if blastocysts did not develop, one or two morula were transfered, whereas lower-cell embryos were not transfered. Biochemical pregnancy was confirmed by the positive serum β-hCG test (> 48 mIU/ml) 15 days after the embryo transfer, and clinical pregnancy by an ultrasound scan of the gestational sac and the embryo heart beats 14 days after the positive β-hCG test.

### Statistics

To evaluate the role of sperm selection by their morphology, the laboratory and clinical outcomes between the IMSI and ICSI groups of couples were compared. The SPSS (Statistical Package for the Social Science; SPSS Inc., Chicago, IL, USA) statistical program for Microsoft Windows was used for statistical calculations. The laboratory outcomes were expressed as fertilization rate (number of fertilized oocytes per injected oocytes), number of blastocysts (%), number of blaststocysts per cycle, and number of cycles with at least one blastocyst (%). The clinical outcomes were expressed as implantation rate (number of implanted embryos per transferred embryos), number of pregnancies, pregnancy rate per cycle, and number of spontanous abortions. For comparing categorical data, the chi-square (χ^2^) test was performed, and Spearman's rank correlation coefficient was used as a non-parametric measure of statistical dependence between the two variables. A probability value (p value) less than 0.05 was considered statistically significant.

## Results

During an 8-month period a total of 57 couples were involved in 20 IMSI cycles (*IMSI group*) and 37 ICSI cycles (*ICSI group*). Both groups contained approximately the same proportion of couples with indications of male infertility combined with indications of female infertility (60.0% and 64.8%). The mean female age in the *IMSI group *was 35.8 years (min. 27 - max. 40 years) and in the *ICSI group *was 32.9 years (min. 25 - max. 42). The mean sperm concentrations in the *IMSI *and *ICSI groups *were 51.3 × 10^6^/ml and 50.2 × 10^6^/ml, the mean sperm motilities were 53.5% and 49.1%, and the mean sperm morphologies 10.8% and 10.7% of normal sperm.

In all IMSI cycles it was possible to get at least one *Class I *or *Class II *sperm. *Class I *sperm were found in 8 (40%) patients; in the other 12 patients only *Class II *sperm were possible to find and inject into the oocytes. In all, 21 of 121 (17%) *Class I *sperm were found.

For the microinjection of oocytes, the average values of the *IMSI *and *ICSI group *outcome parameters are shown in Table 1. A total of 356 MII oocytes were injected by the IMSI (n = 121) and ICSI (n = 235). No significant differences in the fertilization rates (IMSI: 51.2%; ICSI: 52.7%, p = 1.795) and the proportions of triploid zygotes (IMSI: 0%; ICSI: 1.2%, p = 0.393) were observed.

**Table 1:** Results of the IMSI vs. ICSI procedure in couples with poor embryo development - all embryos arrested following a prolonged 5-day culture in previous cycles of ICSI.

	Method of *in vitro *fertilization			
IVF outcome parameters	*IMSI group*		*ICSI group*	
	previous ICSI	current IMSI	previous ICSI	current ICSI
No. of couples	20	20	37	37
Average female age (years)	34.7 ± 4.02	35.8 ± 4.36	32.0 ± 4.04	32.9 ± 4.48
No. of injected oocytes (%)	158 (74.9%)	121 (75.1%)	227 (80.2%)	235 (79.3%)
No. of fertilized oocytes (zygotes) (%)	86 (54.4%)	62 (51.2%)	129 (56.8%)	124 (52.7%)
No. of triploid zygotes (%)	3 (1.9%)	0 (0%)	3 (1.1%)	3 (1.2%)
Average no. of embryos per cycle	2.6 ± 6.21	3.1 ± 3.45	2.8 ± 6.94	3.3 ± 3.29
No. of blastocysts (%)	0 (0%)	16 (26.0%)	0 (0%)	24 (19.3%)
No. of blastocysts pr cycle	0	0.80	0	0.65
No. of arrested embryos - morula (%)	26 (30.2%)	17 (27.4%)	41 (31.7%)	25 (19.1%)
No. of embryos arrested prior to the morula stage (%)	44 (51.2%)	29 (47.0%)	88 (68.2%)	75 (60.5%)
No. of cycles with at least one blastocyst (%)	0 (0%)	10 (50.0%)	0 (0%)	13 (35.1%)
No. of cycles with all embryos arrested (%)	33 (100%)	0 (0%) ^a^	46 (100%)	10 (27.0%) ^b^
Implantation rate	0%	17.1% (6/35)	0%	6.8% (3/44)
No. of pregnancies	0	5	0	3
Pregnancy rate per cycle	0%	25.0%	0	8.1%

Values are average ± SD

a vs. b statistically significant difference as evaluated by the chi-square test (p < 0.05)

In the *IMSI group*, a higher number of blastocysts developed per cycle than in the *ICSI group *(0.80 vs. 0.65). In the *IMSI group*, 26.0% of embryos developed to the blastocyst stage after the IMSI procedure, whereas in the previous ICSI cycles all embryos were arrested at the lower developmental stages. In the *ICSI group*, 19.3% of embryos developed to the blastocyst stage, whereas in the previous ICSI cycles all embryos were developmentally arrested. There was a tendency toward a higher number of cycles with at least one blastocyst in the current IMSI group than current ICSI group (50% and 35.1%, p = 0.750, respectively); although, it was not statistically significant (Table 1).

When blastocysts developed, they did not differ in their quality according the procedure of sperm microinjection, IMSI or ICSI. There were approximately the same proportions of early (25.0% and 29.2%), developing (18.7% and 8.3%), developed (18.7% and 29.2%) and expanded (37.5% and 33.3%) blastocysts in current *IMSI *and *ICSI group *cycles. Hatching or hatched blastocysts were not observed regardless of the group of couples. In both *IMSI *and *ICSI groups *there were also approximately the same proportions of blastocysts with good (33.3% and 35.3%), worse (33.3% and 41.2%), poor (25% and 17.6%), and very poor (8.3% and 5.9%) morphology of the ICM or TE.

In *IMSI group*, 18 IMSI-derived blastocysts were transferred in 10 (50%) cycles (1.8/cycle) and 3 pregnancies were achieved (Table 2). The clinical pregnancy rate per blastocyst transfer was 30%. In the rest of the 10 IMSI cycles, 17 morula embryos were transferred (1.7/cycle) into the uterus. Two pregnancies were achieved thus giving a 20% pregnancy rate per morula transfer.

**Table 2:** Clinical outcome of IMSI and ICSI procedures according to the developmental stage of the transferred embryos in comparison with previous ICSI cycles

	Method of *in vitro *fertilization			
	*IMSI group*		*ICSI group*	
	previous ICSI	current IMSI	previous ICSI	current ICSI
	**Blastocyst transfers**			
No. of transfers	0	10	0	13
No. of transferred embryos	0	18	0	24
Average no. of transferred embryos per transfer	0	1.8 ± 0.29	0	1.8 ± 0.70
No. of pregnancies	0	3	0	3
Pregnancy rate per transfer	0%	30%	0%	23.0%
Spontaneous abortions	0	0	0	2
	**Morula transfers**			
No. of transfers	21	10	35	14
No. of transferred embryos	26	17	41	20
Average no. of transferred embryos per transfer	1.2 ± 0.82	1.7 ± 0.34	1.2 ± 0.75	1.4 ± 0.7
No. of pregnancies	0	2	0	0
Pregnancy rate per transfer	0%	20%	0%	0%
Spontaneous abortions	0	1	0	0
	**Cycles with no embryo transfer due to developmental arrest**			
No. of cycles	12 (36.0%)	0 (0%) ^a^	11(24.0%)	10 (27.0%) ^b^

Values are average ± SD

a vs. b statistically significant difference as evaluated by the chi-square test (p < 0.05)

In the *ICSI group*, 24 ICSI-derived blastocysts were transferred in 13 (35.1%) cycles (1.8/cycle) and 3 pregnancies were achieved. The clinical pregnancy rate per blastocyst transfer was 23% and tended to be lower than in the *IMSI group *(23% vs. 30%, p = 0.180), although not statistically significant. All pregnancies achieved by the blastocyst transfer were singleton and two pregnancies terminated with a spontaneous abortion. In 14 ICSI cycles, at least one morula was transferred into the uterus but no pregnancies were achieved (Table 2).

There was a significantly lower proportion of cycles with all embryos arrested and a consequently lower proportion of cycles with no embryo transfer in the current *IMSI group *compared to the current *ICSI group *(0% vs. 27.0%; p = 0.048; 0%), as shown in Table 2.

The overall implantation and pregnancy rates (following blastocyst and morula transfers) in the current IMSI cycles of the *IMSI group *tended to be higher than in the current ICSI cycles of the *ICSI group *(17.1% vs. 6.8%, p = 1.162; 25.0% vs. 8.1%; p = 1.829, respectively), as shown in Table 1 although not statistically significant.

In terms of pregnancy, all pregnancies achieved by the blastocyst transfer in the IMSI group were singleton and normally on-going, whereas two from these pregnancies achieved by the blastocyst transfer in the ICSI group terminated with a spontaneous abortion. After IMSI-derived morula transfer, one pregnancy terminated with a spontaneous abortion and another was a normally on-going singleton pregnancy.

## Discussion

The results of this study have shown that the IMSI procedure is a method of choice in infertile couples with indications of male infertility and with poor embryo development in previous ICSI attempts. Couples with all embryos arrested after a prolonged embryo culture of 5 days are relatively rare and represent the most difficult cases in the *in vitro *fertilization programme; therefore, the studied groups in our study are relatively small. The benefits of IMSI to improve outcomes of male infertility patients have already been published [7,8,13,14]. However, the data about the improvement in laboratory and clinical outcomes when comparing ICSI and IMSI in the same couples is new.

Similar to our study, Vanderzwalmen et al. (2008) observed in a sibling oocyte study including 20 infertile couples that sperm selection at high magnification before ICSI, according to the morphology and head vacuoles, improves embryo development to the blastocyst stage. They divided sperm into 4 categories and concluded that optimal sperm without vacuoles or with 1 or 2 small vacuoles in their heads produced significantly higher rates of blastocysts (56.3 and 61.4%, respectively) than sperm with large head vacuoles or large head vacuoles combined with other morphological abnormalities (5.1 and 0%, respectively) [13]. Cassuto et al. (2009) observed that the IMSI procedure improved fertilization and blastocyst rates in 27 infertile couples [14].

In patients included in this study, the mean sperm counts (51.3 and 50.2 × 10^6^/ml respectively) were reasonable and the mean morphologies (10.8% and 10.7% respectively) were within the original Kruger classification of "subfertile/fertile" with good prognosis. It is known that normal classical parameters of sperm quality are not always related to fertility and good prognosis, because sperm might have some other functional abnormalities, i.e. sperm DNA fragmentation and head vacuoles. Therefore the IMSI procedure possibly improved the laboratory and clinical outcome of sperm microinjection in this group. Twenty-four blastocysts were also transferred in the regular ICSI group in the current study. This might also reflect the fact that sperm quality was possibly improved; it is known that the sperm quality is not a constant but may vary. It should also be stressed that in a relatively high proportion of couples of both groups, indications of male infertility were combined with indications of female infertility, and therefore the female effect on the laboratory and clinical outcome of sperm microinjection could not be completely excluded.

It seems that the positive effect of sperm selection at high magnification is expressed at later stages of embryo development to the blastocyst stage. In the present study there was no significant positive effect of the IMSI procedure on the fertilization of the oocytes and abnormal triploid fertilization. Mauri et al. (2010) found that IMSI did not improve the quality of day 2 embryos in comparison with ICSI [19] in a sibling oocyte study. Similarly, Vanderzwalmen et al. found that IMSI provided no significant difference in embryo quality up to day 3 but led to a better development of embryos to the blastocyst stage when compared to conventional ICSI [13]. IMSI seems to positively affect embryo development after the activation of the embryonic genome and represents a late paternal positive effect, which is associated with an increased incidence of sperm DNA fragmentation [20]. IMSI improves the embryo development to the blastocyst stage via the selection of sperm without vacuoles in their heads, which is influenced by the incidence of sperm DNA fragmentation [10,11].

Our results showed improved embryo development and quality after the IMSI procedure reflected in a higher number of blastocysts per cycle and a significantly lower proportion of cycles with no embryo transfer due to developmental arrest in comparison with the conventional ICSI procedure. There was also a tendency toward a higher blastocyst rate per cycle, a lower proportion of arrested embryos (i.e. less than the morula stage by day 5 of the culture), and a lower proportion of cycles with all embryos arrested after a prolonged 5-day culture in comparison with ICSI procedure. In current *IMSI cycles*, the implantation and pregnancy rates tended to be better than in current ICSI cycles, in spite of a tendency to a higher mean female age. IMSI seems to be promising for older women but needs to be confirmed in research with a larger number of couples.

Embryo culture, especially prolonged embryo culture to the blastocyst stage (5 days), can serve as a strong diagnostic tool [21] reflecting indications of male and female infertility and yielding useful information regarding the implantation potential of the human embryo. In this study, there was no significant difference in the pregnancy rates after the IMSI and ICSI procedures after blastocyst transfer, but after ICSI two out of three pregnancies terminated by spontaneous abortion, whereas after IMSI there were no spontaneous abortions. This shows that the IMSI procedure might provide more "healthy" blastocysts without chromosomal abnormalities, possibly, in spite of very comparable development and morphology to ICSI-derived blastocysts. From the literature it is known that embryos with normal morphology may also be chromosomally abnormal and can develop to the blastocyst stage [22]. This was also true for the morula transfer, when there was no blastocyst on day 5. It is interesting that morula also implanted successfully after the IMSI procedure, whereas ICSI-derived morula did not implant.

The results of this research showed that by IMSI it is possible to improve the laboratory and clinical outcome of *in vitro *fertilization in couples with previous poor embryo development.

## Conclusions

We can conclude that sperm selection at high magnification using differential interference contrast (DIC), immersion objective, and a digital camera is useful in identifying more precisely the sperm morphology and the size and the number of sperm head vacuoles that exert a considerable negative effect on embryo development to the blastocyst stage, and consequently on the pregnancy. By using the IMSI procedure the embryo development and pregnancy might be improved in couples with indications of male infertility, poor sperm quality and poor embryo development, i.e. on all embryos arrested following a prolonged 5-day culture in previous conventional ICSI attempts.

## Competing interests

The authors declare that they have no competing interests.

## Authors' contributions

KK has performed all IMSI and ICSI procedures in the frame of her Ph.D and writed this manuscript under supervision of IVK. BZ recruited all of the patients included in the study. TT and EBV performed all ovarian hormonal stimulations in couples included in the study. IVK supervised KK and made corrections to this manuscript. All authors read and approved the final manuscript.
